# COVID-19 intermittent spikes in Pakistan and reluctancy towards vaccination due to lack of education- an incipient crisis

**DOI:** 10.1016/j.amsu.2022.103418

**Published:** 2022-03-10

**Authors:** Ubaid Khan

**Affiliations:** Department of Medicine, King Edward Medical University Lahore, Pakistan

To The Editor,

The world has shifted in a new direction since December 2019 when the coronavirus breakout shook the core of this Earth. In Pakistan, on Feb 20, 2020, the first case of COVID-19 was reported and currently, a number of COVID-19 cases have been reached 1.47 million (Fig. 1) [1] (see Fig. 2).

**Fig. 1:**
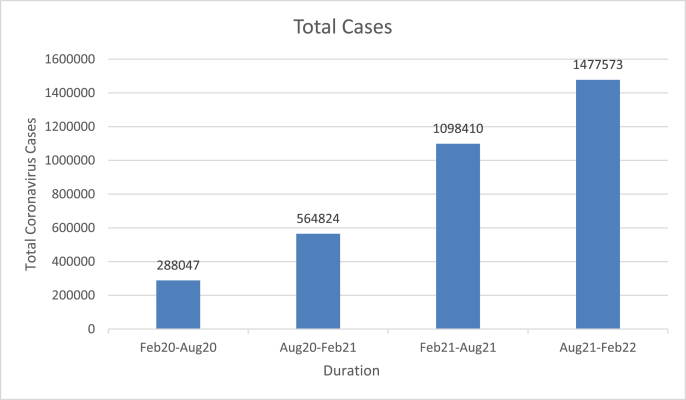
Total no of cases.

**Fig. 2:**
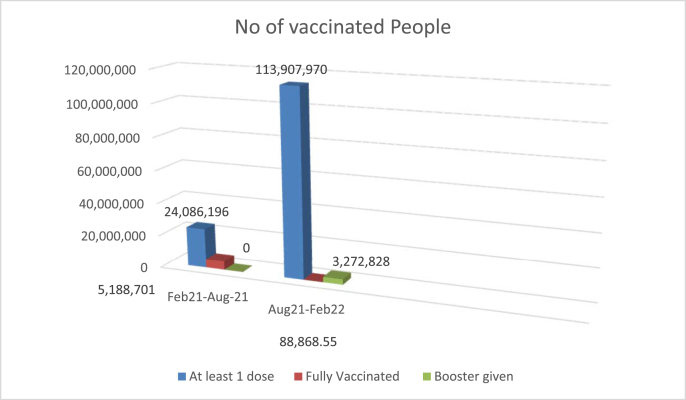
Statistics of vaccination administration with respect to time.

So far, Pakistan has faced five waves of COVID-19. The initial surge in Coronavirus cases was because people did not follow Standard Operating procedures and the current spikes in coronavirus cases is due to inadequate implementation of SOPS and the people's unwillingness to vaccination. This Letter is meant to throw light on different factors contributing to the cause of Covid-19 spread in Pakistan.

COVID-19 is a lethal disease caused by enveloped positive-strand mRNA virus. According to World Health Organization (WHO), about 5.79 million deaths have been reported globally due to COVID-19 [1]. Corona Virus is highly contagious and it directly affects the respiratory tract, causing nasal obstruction, dry cough, weakness, loss of taste and smell. Direct invasion of corona virus damages the respiratory tract with visible sclerosis [2]. Re-infection rate is also high. Despite these concerns and government efforts, only 40.7% percent of the Pakistani population is fully vaccinated against the virus compared to other countries in which the rate of vaccination is 87.0% in China, 71.4% in Brazil, and 64.6%. in the United States, respectively [3].

Additionally, according to the NCOC of Pakistan, a total of 194 million vaccine doses have been administrated, out of which 113,907, 970 people have been given the first dose of vaccine and 88,868,55 people are fully vaccinated against the disease [4]. This decline in the rate of vaccination in Pakistan is due to a lack of education, awareness programs, and conspiracy theories about coronavirus and vaccines. For example, some people claimed the virus is a big illusion created to target Islamic nations, allowing Jews to take over the world and using nano-chips embedded in people's bodies to obtain control via 5G towers [5].

These theories are actively discussed on social media in the Pakistani community. In a country where vaccine hesitancy is a significant obstacle to eradicate vaccine-preventable diseases, such conspiracy tales may lay the foundations of resistance against COVID-19 vaccination programs. Additionally, people also believe that vaccine is harmful and will have deteriorating effects on their health. A survey was conducted to check the general perception of the people about the Covid −19 vaccine. This study indicated that 52% of the participants were concerned about the vaccine's side effects. Moreover, 72% of people planned to get the vaccine, whereas 28% refused to get the vaccine [6]. Therefore, awareness programs are required to address the public about the safety and need of the coronavirus vaccine.

## Ethical approval

N/A.

## Sources of funding

This research did not receive any specific grant from funding agencies in the public, commercial, or not-for-profit sectors.

## Author contributions

Ubaid khan is the only author.

## Trial registry number

Not applicable.

## Guarantor

Ubaid Khan.

## Consent

N/A.

## Provenance and peer review

Not commissioned, Editor reviewed.

## Declaration of competing interest

There is no conflict of interest.

## References

[1] WHO Covid 19 Statistics (2021). https://covid19.who.int/.

[2] Ghosh B., Sarkar S., Sepay N., Das K., Das S., Dastidar S.G. (2021). Factors for COVID-19 infection that govern the severity of illness. SciMedicine Journal.

[3] Hannah Ritchie E.M., Rodés-Guirao Lucas, Appel Cameron, Giattino Charlie, Ortiz-Ospina Esteban, Joe Hasell, Macdonald Bobbie, Beltekian Diana, Roser Max (2020).

[4] Pakistan NGO.

[5] Staff OJOAahwocc-pm-c-t-c-i-u-u-c-c-cAJ (2020).

[6] Yasmin F., Asghar W., Babar M.S., Khan H., Ahmad S., Hameed Z. (2021). Acceptance rates and beliefs toward COVID-19 vaccination among the general population of Pakistan: a cross-sectional survey. Am. J. Trop. Med. Hyg..

